# Evidence for the Possible Biological Significance of the *igf-1* Gene Alternative Splicing in Prostate Cancer

**DOI:** 10.3389/fendo.2013.00031

**Published:** 2013-03-20

**Authors:** Anastassios Philippou, Athanasios Armakolas, Michael Koutsilieris

**Affiliations:** ^1^Department of Experimental Physiology, Medical School, National and Kapodistrian University of AthensAthens, Greece

**Keywords:** apoptosis, cancer progression, cell survival, human IGF-I isoforms, IGF-I bioactivity, IGF-I peptides, IGF-I receptors, IGF-I signaling

## Abstract

Insulin-like growth factor-I (IGF-I) has been implicated in the pathogenesis of prostate cancer (PCa), since it plays a key role in cell proliferation, differentiation, and apoptosis. The IGF-I actions are mediated mainly via its binding to the type I IGF receptor (IGF-IR), however IGF-I signaling via insulin receptor (IR) and hybrid IGF-I/IR is also evident. Different IGF-I mRNA splice variants, namely IGF-IEa, IGF-IEb, and IGF-IEc, are expressed in human cells and tissues. These transcripts encode several IGF-I precursor proteins which contain the same bioactive product (mature IGF-I), however, they differ by the length of their signal peptides on the amino-terminal end and the structure of the extension peptides (E-peptides) on the carboxy-terminal end. There is an increasing interest in the possible different role of the IGF-I transcripts and their respective non-(mature)IGF-I products in the regulation of distinct biological activities. Moreover, there is strong evidence of a differential expression profile of the IGF-I splice variants in normal versus PCa tissues and PCa cells, implying that the expression pattern of the various IGF-I transcripts and their respective protein products may possess different functions in cancer biology. Herein, the evidence that the IGF-IEc transcript regulates PCa growth via Ec peptide specific and IGF-IR/IR-independent signaling is discussed.

## Introduction

**Figure 1:**
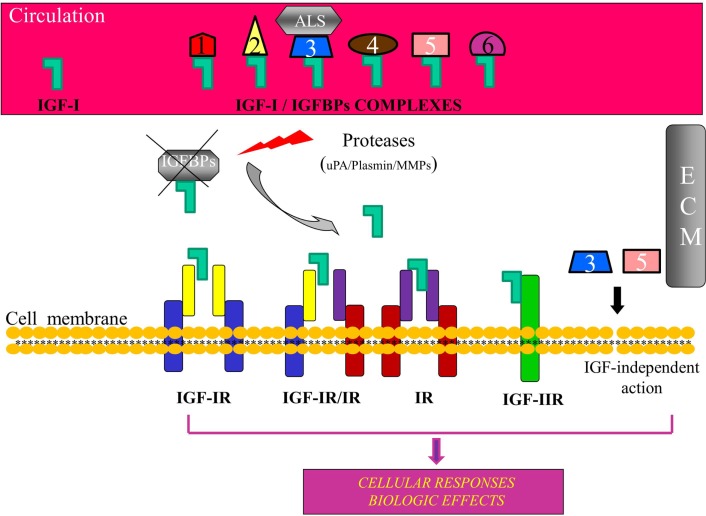
**Schematic representation of the insulin-like growth factor-I (IGF-I) bioregulation system**. Apart from IGF-I, the system consists of the receptors IGF-IR, IGF-IIR, insulin receptor (IR), and IGF-IR, IR hybrids, and at least six high affinity insulin-like growth factor binding proteins (IGFBPs). IGF-I circulates mainly in an IGF/IGFBP-3/ALS complex. Binding of IGFBPs to IGF-I prevents the ligand to interact with the receptor(s) and IGFBPs can modulate, both in the circulation and in the extracellular environment (ECM), the extent of IGF-I-dependent cellular effects. Proteolysis of IGFBPs by proteases, such as urokinase-type plasminogen activator (uPA), plasmin, metalloproteinases (MMPs), and prostate-specific antigen (PSA), results in an increase of bioavailability of IGF-I for interaction with the IGF-IR. Some IGFBPs can exert also an IGF-IR-independent bioactivity. ALS, acid-labile subunit; ECM, extracellular matrix.

**Figure 2:**
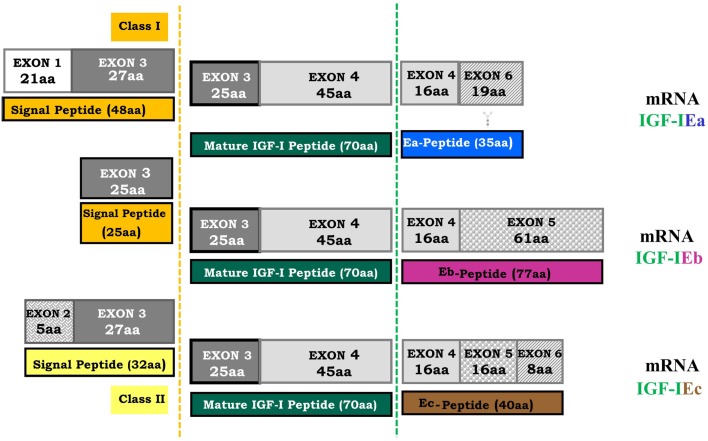
**Human *igf-1* gene alternative splicing**. All possible combinations between leader sequence (signal peptide) usage and terminal exon (5 or 6) can occur in different IGF-I mRNA isoforms. The mature IGF-I peptide is coded by exons 3 and 4. It is a common part of the IGF-I precursor polypeptides and it is derived from post-translational processing of each of the multiple IGF-I precursors, by which the signal and the E-peptides (Ea, Eb, Ec) are removed (dashed lines represent the cleavage sites). The different E-peptides are encoded by three mRNA variants produced by alternative splicing of the 3′ end of the pre-IGF-I mRNA. The first 16 amino acids of the amino-terminal portion of the IGF-I E-peptides are coded by exon 4. Exons 5 and 6 encode, by alternative splicing, distinct portions of the E-peptides with alternative carboxy-terminal sequences. An N-linked glycosylation site, contained only in the Ea-peptide, is also represented (
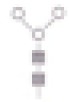
).

A variety of cellular responses are induced by insulin-like growth factor-I (IGF-I), including cell proliferation, differentiation, migration, and survival (Jones and Clemmons, 1995; Reyes-Moreno et al., 1998; Koutsilieris et al., 2000b; Siddle et al., 2001; LeRoith and Roberts, 2003; Kooijman, 2006). These cellular responses have implicated IGF-I in the pathophysiology of several human cancers (Werner and LeRoith, 1996; Werner and Bruchim, 2009). In particular, there is an extensive body of literature suggesting that the IGF system (Figure 1) is importantly involved not only in prostate gland growth and development but also in prostate cancer (PCa) growth and progression (Polychronakos et al., 1991; Reyes-Moreno et al., 1998; Grimberg and Cohen, 1999; Koutsilieris et al., 2000b; Wetterau et al., 2003; Monti et al., 2007; Werner and Bruchim, 2009). Due to alternative splicing of the igf-1 gene, different IGF-I mRNA transcripts are produced encoding several IGF-I precursor proteins (isoforms), i.e., the IGF-IEa, IGF-IEb, and IGF-IEc, which differ by the length of their signal peptides on the amino-terminal end and the structure of their extension peptides (called E domains, or E-peptides) on the carboxy-terminal end (Siegfried et al., 1992; Gilmour, 1994; Chew et al., 1995; Wallis, 2009) (Figure 2).

However, much less is known about the expression profile of the IGF-I splice variants and the potentially distinct biological roles of the IGF-I isoforms in the pathophysiology of various types of cancer (Siegfried et al., 1992; Kuo and Chen, 2002; Armakolas et al., 2010; Koczorowska et al., 2011; Philippou et al., 2011; Kasprzak et al., 2012). Herein, we shall focus on the concept that the differential expression of IGF-I isoforms are possibly involved in the pathophysiology of PCa, presenting evidence that IGF-IEc related peptides may act via an IGF-I receptor (IGF-IR)-independent and insulin receptor (IR)-independent pathway.

## IGF-I mRNA Alternative Splicing

Different leader sequences result in two different classes of IGF-I mRNA isoforms. Class I transcripts use exon 1 as leader exon, whereas class II transcripts have their initiation sites on exon 2. Alternative splicing of exon 5 results also in different mRNA variants containing exon 5 (IGF-I Eb), or containing exon 6 and excluding exon 5 (IGF-I Ea) (Okazaki et al., 1995; Bloor et al., 2001; Barton, 2006). In the human igf-1 gene, alternative splicing generates also a third variant, the IGF-I Ec, which contains both exon 5 and 6 and corresponds to IGF-I Eb in rodents (Chew et al., 1995). More specifically, IGF-I Ea transcript derives from the splicing pattern exon 1 or 2–3–4–6 of the igf-1 gene, which represents the main IGF-I mRNA produced in liver (Jansen et al., 1983; Chew et al., 1995). IGF-I Eb transcript is a splice variant of exon 1 or 2–3–4–5 (Rotwein, 1986), while IGF-I Ec transcript is an exon 1 or 2–3–4–5–6 splice variant of the *igf-1* gene (Chew et al., 1995). Structurally, the IGF-I Ec mRNA transcript differs from the IGF-I Ea variant by the presence of the first 49 base pairs from exon 5 (52 bp in rodents) (Figure 2). The biological significance of IGF-I splice variants is currently unknown, however the presence of different transcripts is indicative of diverse responses to different stimuli (Yu and Rohan, 2000) and they probably reflect the complexity of the IGF-I isoforms-mediated actions in different pathophysiological conditions (Mourkioti and Rosenthal, 2005; Temmerman et al., 2010).

## The Components of the IGF-I Bioregulation System

Insulin-like growth factor-I mediates its actions through binding to specific receptors, such as type I (IGF-IR) and type II (IGF-IIR) IGF receptor, IR, and several atypical receptors, such as the hybrid IGF-IR/IR (Federici et al., 1997; Le Roith et al., 2001; Nakae et al., 2001; Taguchi and White, 2008) (Figure 1). More specifically, IGF-IR binds IGF-I with the highest affinity and also IGF-II and insulin with approximately 10-fold and 100-fold lower affinity, respectively. The IGF-I is also able to interact with the IR, but with much lower affinity (Laviola et al., 2007). IGF-IR exhibits a high degree of homology to IR (De Meyts and Whittaker, 2002) and both IGF-I and insulin can cross-activate these receptors, while the IGF-IR signaling pathways share multiple intracellular mediators with the insulin signaling cascade (Duan et al., 2010). The IGF-IR/IR hybrid receptor binds both insulin and IGF-I, although its binding affinity for insulin is lower than that for IGF-I it has lower affinity for insulin than classical IR, and is thought to function predominantly as an IGF-IR, however the functional importance of IGF-IR/IR hybrid receptor remains poorly understood (Soos et al., 1993; Yu and Rohan, 2000; Taguchi and White, 2008). IGF-IIR binds IGF-II with the highest affinity, IGF-I with much lower affinity, and it does not bind insulin (Laviola et al., 2007).

Biological actions of IGF-I are modulated by a family of at least six insulin-like growth factor binding proteins (IGFBPs) (Oh, 1997; Baxter, 2000; Mourkioti and Rosenthal, 2005; Cohen, 2006). In general, IGFBPs bind IGF-I and increase its half-life both in the extracellular matrix and transfer IGFs in the circulation. Most of the circulating IGF-I is found in a ternary complex with IGFBP-3 and the glycoprotein acid-labile subunit (ALS), and this complex protects IGF-I from proteolytic degradation (Baxter et al., 1989; Yu and Rohan, 2000). IGF-I acts primarily through the binding and activation of IGF-IR (Le Roith et al., 2001; Laviola et al., 2007; Philippou et al., 2007), and ligation of IGF-IR initiates intracellular signaling cascades involved in mitogenic, cell survival, anti-apoptotic, and transforming activities (Krueckl et al., 2004; Tenta et al., 2005a; Samani et al., 2007; Werner and Bruchim, 2009).

Several unique processing features of IGF-I precursor protein have been described, suggesting that post-translational processing is a regulatory mechanism of the IGF-I activity (Duguay et al., 1995; Duguay, 1999). In addition, the Ea-peptide of the human IGF-IEa isoform contains an N-linked glycosylation site and this glycosylation might also play a role in regulation of the bioavailability of the mature IGF-I (Duguay, 1999; Hede et al., 2012). The IGF-I domain which is responsible for the binding of the IGF-IR is the mature IGF-I peptide (Figure 2); it is a biologically active product derived from post-translational processing of each of the multiple IGF-I precursor polypeptides (Rotwein et al., 1986; Barton, 2006; Wallis, 2009). After post-translational cleavage of the pro-IGF-I isoforms, the IGF-I E-peptides are proteolytically removed (Rotwein et al., 1986; Duguay et al., 1995; Denley et al., 2005; Shavlakadze et al., 2005). It has been previously proposed that they are also biologically active and elicit functions that are distinct to the mature IGF-I in bronchial epithelial and neuroblastoma cells (Siegfried et al., 1992; Kuo and Chen, 2002).

Thus, a growing interest has arisen with regard to the aspect of the differential expression of IGF-I mRNA isoforms or respective post-translational E-peptides actions in various *in vitro* (Mills et al., 2007; Pfeffer et al., 2009; Quesada et al., 2011; Brisson and Barton, 2012) and *in vivo* (Dluzniewska et al., 2005; Philippou et al., 2009; Stavropoulou et al., 2009; Armakolas et al., 2010; Barton et al., 2010; Gentile et al., 2010; Milingos et al., 2010) models and pathologies. Furthermore, there has been increasing focus on the potential of differential IGF-I isoforms actions through a putative E-peptide-specific signaling (Philippou et al., 2009; Quesada et al., 2009; Stavropoulou et al., 2009; Deng et al., 2011; Brisson and Barton, 2012) and particularly in PCa (Armakolas et al., 2010).

## IGF-I System and Its Involvement in PCa Biology

### IGF-I actions in PCa

An intricate balance between cell proliferation factors and apoptosis-inducing and apoptosis-inhibiting factors is critical in regulating prostate growth (Reyes-Moreno et al., 1998; Reynolds and Kyprianou, 2006; Tenta et al., 2007; Pitulis et al., 2009). Disruptions in the balance between apoptotic and cell growth factors is a mechanism that triggers the evasion of apoptosis and the over-expression of factors that promote cell proliferation and survival leading, thus, to carcinogenesis and cancer progression (Reyes-Moreno et al., 1998; Reynolds and Kyprianou, 2006). In particular, during PCa progression, PCa biology is characterized by blockade of apoptosis (survival), uncontrolled proliferation and increased invasive and metastatic potential (Mitsiades et al., 2000; Reynolds and Kyprianou, 2006).

The IGF system has been implicated in several human cancers (Werner and LeRoith, 1996) and a significant amount of data suggests that it plays an important role in PCa initiation and progression (Werner and Bruchim, 2009). Specifically, IGF-I exerts a highly mitogenic and anti-apoptotic activity in cells (Wu et al., 2001) and the relative contribution of endocrine versus tissue IGF-I in growth control has been an essential question (Le Roith et al., 2001). Several prospective studies have suggested that high circulating IGF-I levels were associated with increased mitogenic and anti-apoptotic effects and an increased risk of developing PCa (Chan et al., 1998; Grimberg and Cohen, 1999; Khosravi et al., 2001; Monti et al., 2007; Werner and Bruchim, 2009). In addition, meta-analysis studies have shown that IGF-I is significantly associated with a high relative risk for developing PCa (Harman et al., 2000; Shaneyfelt et al., 2000) and that PCa patients had significantly higher circulating levels of IGF-I (Stattin et al., 2000; Shi et al., 2001). Thus, the positive correlation between high circulating IGF-I levels and PCa progression has implicated IGF-I as an etiologic factor of PCa (Stattin et al., 2004; Reynolds and Kyprianou, 2006). However, there is also evidence that does not support a causal association between serum IGF-I (or IGFBP-3, discussed below) and the risk of PCa (Woodson et al., 2003), and it was hypothesized that high-grade PCas are more autonomous and less sensitive to the action of IGF-I than low-grade cancers (Nimptsch et al., 2011). Moreover, the results from a recent study, which provides the largest assessment of the role of the IGF system in the development of prostate-specific antigen (PSA)-detected PCa, suggested that circulating IGF-I has a limited role in the development of early PCa but it may remain an important risk factor for disease progression (Rowlands et al., 2012). Furthermore, IGFBP-3 levels have been inversely associated with prostate carcinogenesis and the negative correlation between IGFBP-3 levels and cancer risk is consistent with a protective role of IGFBP-3, i.e., high IGFBP-3 concentrations may lead to reduced IGF-I bioavailability (Koutsilieris et al., 1995; Bogdanos et al., 2003; Papatsoris et al., 2005; Werner and Bruchim, 2009).

The deregulation of growth factors activity, such as of IGF-I (Koutsilieris, 1993; Koutsilieris et al., 2000b), transforming growth factor-β (TGF-β) (Koutsilieris, 1993), urokinase-type plasminogen activator (uPA) (Koutsilieris, 1993; Koutsilieris et al., 2000b), and basic fibroblast growth factor (bFGF), have an important role in PCa disease progression (Koutsilieris et al., 1990) and castration-resistant growth mainly in bone (Reyes-Moreno et al., 1998; Mitsiades et al., 2000; Karamanolakis et al., 2002; Bogdanos et al., 2003; Katopodis et al., 2009) and lymph nodes metastasis (Koutsilieris et al., 1986, 1987; Sourla et al., 1996; Reynolds and Kyprianou, 2006). In particular, PCa cells that have metastasized to bone have an upregulated IGF-I regulatory system (Ozkan, 2011). Thus, the growth factor signaling pathways that regulate apoptosis and proliferation offer significant molecular targets for therapeutic intervention of castration-resistant PCa (Bogdanos et al., 2003; Reynolds and Kyprianou, 2006; Sachdev and Yee, 2007), and such interventions include anti-IGF-I therapy (Koutsilieris et al., 2000a; Tenta et al., 2004), anti-survival factor therapy (Koutsilieris et al., 2000b), anti-bone microenvironment-related growth factors therapy (Tenta et al., 2004, 2005b), or a dexamethasone and somatostatin analog combination therapy (Koutsilieris et al., 2001; Dimopoulos et al., 2004).

### IGF-I/IGF-IR/IGFBPs system in PCa

Previous studies have focused on the use of *in vitro* primary cell cultures in order to characterize the expression of IGF-I separately in each cellular compartment of the prostate; the initial findings suggested that prostatic epithelial cells, whether from normal, benign prostatic hyperplasia, or malignant tissues, do not synthesize or secrete significant amounts of IGF-I (Peehl et al., 1996). However, established PCa cell lines such as PA-III, PC-3, LNCaP, and DU145, have been shown to express IGF-IR and sometimes IGF-I (Polychronakos et al., 1991; Nickerson et al., 2001; Kawada et al., 2006; Armakolas et al., 2010). In benign prostatic tissue, IGF-I expression was observed only in a small percentage and at a weak staining intensity in the tissue, while high-grade tumor showed a stronger reaction (Ozkan, 2011). Also, staining in epithelial cells of both prostatic intraepithelial neoplasia (PIN) and invasive tumors confirmed that neoplastic epithelial cells and also PIN express IGF-I (Ozkan, 2011).

Regardless of the extent of IGF-I secretion, PCa cells do express IGF-IR (Peehl et al., 1996). In addition, it has been demonstrated that over-expression of IGF-IR can potentiate tumor growth and can behave like an oncogene (Kaleko et al., 1990; Polychronakos et al., 1991; Ozkan, 2011). There is much evidence showing the relationship of IGF-IR and its ligands with the development and progression of PCa (Baserga et al., 1997; Hellawell and Brewster, 2002; Hellawell et al., 2002), which has been summarized as follows: IGF-IR plays a important role in cellular transformation, it has a critical role in the protection of cells from apoptosis, and its activation or over-expression mediates many aspects of malignant phenotype-like metastatic potential (Ozkan, 2011). IGF-IR-induced cell growth and survival are both conductive to increased tumor growth, while inversely, down-regulation of the IGF-IR leads to apoptosis of tumor cells and inhibition of tumor growth (Baserga et al., 2003). IGF-IR is expressed in normal prostate tissue, benign hyperplasia, neoplastic prostate tissues and metastases, as well as in cultured cell lines (Djakiew, 2000; Ryan et al., 2007). Specifically, it is more strongly expressed in epithelial malignant cells than PIN and normal cells, acting as an autocrine signal to the epithelial compartment (Cardillo et al., 2003). A more intense staining for IGF-IR has been also reported in the stromal tissue surrounding the tumor compared with the surrounding benign tissue (Ryan et al., 2007). Nevertheless, there are studies that did not find appreciable differences in IGF-IR levels between normal prostate and PCa, or showed a decreased IGF-IR expression in primary tumors compared with benign tissues (Chott et al., 1999; Dhanasekaran et al., 2001; Werner and Bruchim, 2009). Interestingly, during transformation of prostate epithelial cells from a benign to a metastatic state, a marked reduction in IGF-IR levels has been reported (Plymate et al., 1997; Chott et al., 1999).

Both normal prostate epithelial cells and PCa cells exhibit IGF-I responsiveness *in vitro* (Cohen et al., 1991; Peehl et al., 1996; Nickerson et al., 2001). Moreover, over-expression of human IGF-I in prostate epithelial cells in a transgenic mouse model led to activation of the IGF-IR and spontaneous tumorigenesis in prostate epithelium (DiGiovanni et al., 2000), while suppression of IGF-IR inhibited prostate tumor cell growth and invasion in rats (Burfeind et al., 1996). In addition, it has been shown that IGF-I from the prostate stromal cells mediates the tumoral stromal cell growth and accelerates tumor growth in the prostate (Wang and Wong, 1998; Kawada et al., 2006). IGF-I, produced by prostatic stromal cells in response to androgen stimulation, has been shown to act in a paracrine manner, stimulating the surrounding prostatic epithelial cells, and resulting in increased proliferation and prostatic carcinogenesis (Wang and Wong, 1998; Bogdanos et al., 2003; Reynolds and Kyprianou, 2006). Stromal cells also express IGF-IR and therefore it has been proposed that they should be presumably responsive to the mitogenic activity of IGF-I (Peehl et al., 1996; Ozkan, 2011). Thus, targeting the igf-1 gene in the prostatic stromal cells has emerged as a potentially attractive modality for treating PCa (Reynolds and Kyprianou, 2006).

IGF-I receptor has been suggested to play an important role not only in PCa progression but also, possibly, in the progression to castration-resistant disease (Wu et al., 2006). Receptor kinases are important determinants of neoplastic behavior (Blume-Jensen and Hunter, 2001) and it was suggested that expression of genes related to receptor tyrosine kinase systems, such as the IGF-I/IGF-IR, may confer castration-resistance (Nickerson et al., 2001; Mitsiades et al., 2006). By using *in vitro* model(s) which mimics events that occur during the natural progression of PCa, i.e., the androgen dependence to androgen independence transition, it was indicated that increased IGF-IR expression is associated with androgen-independent anti-apoptotic and mitotic IGF signaling in the progression of PCa (Nickerson et al., 2001; Krueckl et al., 2004).

Interactions between the IGF-I/IGFBPs bioregulation system pathways and other pathways can modulate the development and progression of PCa (Reyes-Moreno and Koutsilieris, 1997; Reyes-Moreno et al., 1998; Reynolds and Kyprianou, 2006). In normal cells, the IGF-I pathway is inhibited by the IGFBPs since they bind to IGF-I and prevent pathway activation through the interaction of IGF-I with its receptor, while about 99% of the free IGF is bound to IGFBPs and mostly to IGFBP-3 (Djavan et al., 2001; Stewart and Weigel, 2005; Reynolds and Kyprianou, 2006). A direct correlation has been demonstrated between the inhibition of IGF-IR gene expression and up-regulation of IGFBP-3 in the androgen-independent PC-3 cells, while this inhibition led to inhibition of cell proliferation and invasion, and to enhanced spontaneous apoptosis, indicating an important role for both IGF-IR and IGFBP-3 in the homeostasis of prostate carcinoma cells (Grzmil et al., 2004). In addition, activation of TGF-β signaling pathway in the normal prostate induces the up-regulation of IGFBP-3 expression, which could lead to the binding of IGFBP-3 with any excess IGF-I, thus preventing the activation of the IGF-I growth and survival pathways (Koutsilieris, 1995; Nickerson et al., 1997; Bogdanos et al., 2003). Conversely, dysfunction of the TGF-β signaling can lead to increased activation of the IGF-I pathways, eventually leading to tumorigenesis (Reyes-Moreno and Koutsilieris, 1997; Reyes-Moreno et al., 1998; Reynolds and Kyprianou, 2006). It has been shown that IGFBP-3 can block IGF-induced proliferation of prostatic epithelial cells in culture, while the addition of PSA restored proliferation by proteolytic cleavage of IGFBP-3, thus freeing IGF-I for interaction with IGF-IR (Cohen et al., 1994) (Figure 1).

### IGF-I mRNA isoforms in PCa

The data from *in vivo* and *in vitro* studies reviewed above, regarding the IGF system components and their role in the development and progression of PCa, show a differential expression profile during the transformation of prostate epithelial cells from a benign to malignant or metastatic state. It remains to be verified whether those differential profiles reflect different regulatory roles of the IGF-I system components during the transition of the normal prostate tissue to a precancerous or malignant state. Interestingly, a differential expression particularly of the IGF-I mRNA isoforms has been recently documented in human normal and PCa tissues, as well as in human androgen-independent (PC-3) and androgen-dependent (LNCaP) cells (Armakolas et al., 2010). Specifically, the IGF-IEc isoform was found to be expressed, not only at the mRNA but also, by using an IGF-IEc specific antibody (Philippou et al., 2008), at the protein level, in PCa tissues and in the cancer PC-3 and LNCaP cells. Moreover, the expression/localization of this isoform was remarkably higher in PCa and PIN than in normal prostate tissues (Armakolas et al., 2010). Normal human prostate epithelial cells (HPrEC) did not express IGF-IEc transcript (Armakolas et al., 2010).

A differential expression profile of the IGF-I isoforms between normal and cancerous tissues has been also observed in other human cancers *in vivo*, such as in cervical (Koczorowska et al., 2011) and colorectal cancer (Kasprzak et al., 2012), and in osteosarcoma cells *in vitro* (Philippou et al., 2011). Similarly, a differential IGF-I isoforms expression has been found in other human pathologies, such as in skeletal muscle after exercise-induced damage (Philippou et al., 2009) and in endometriosis (Milingos et al., 2010), implying the potentially different roles of the IGF-I isoforms in the pathophysiology of all those conditions. Interestingly and particularly in cancer, IGF-I splice variants appear to be sensitive to the specific cancer type and the state of the disease, showing a differential regulation of specific isoform(s) (Armakolas et al., 2010; Koczorowska et al., 2011; Kasprzak et al., 2012).

Furthermore, it was documented that a synthetic Ec peptide, which comprised the region beyond the common sequence of the human E domains (i.e., a synthetic peptide similar to the C-terminal 24-residues of the human Ec domain), possesses bioactivity in PCa cells. This activity was shown to be mediated possibly via an IGF-IR-independent and IR-independent mechanism, not only in the PCa cells PC-3 and LNCaP (Armakolas et al., 2010) but also in MG-63 osteosarcoma cells (Philippou et al., 2011) and in KLE endometrial-like cells (Milingos et al., 2010). Specifically, the mitogenic action of the synthetic Ec peptide on these human cell lines, induced after its exogenous administration, was not blocked by either a neutralizing anti-IGF-IR antibody or the siRNA knock-out of IGF-IR or IR, which are involved in the IGF-I-mediated actions. At the same time, IGF-I action on these cells was completely abolished (Armakolas et al., 2010; Milingos et al., 2010; Philippou et al., 2011). Hence, it was concluded that the proliferative activity of the synthetic Ec peptide is not propagated through the IGF-IR, IR, or the hybrid IGF-IR/IR receptor (Armakolas et al., 2010).

Similarly, unique and autonomous activities of synthetic human Eb peptide(s) have been also reported, regulating growth and differentiation of human normal and malignant bronchial epithelial cells, as well as neuroblastoma cells, potentially through binding to putative membrane receptor sites distinct from those for IGF-I and insulin (Siegfried et al., 1992; Kuo and Chen, 2002, 2003). In addition, an antitumor activity of human Eb peptide, in terms of inhibition of cell growth and invasion, and of angiogenesis, has also been reported for human breast cancer cells (Chen et al., 2007). The possible IGF-IR- and IR-independent action particularly of the synthetic human Ec peptide was also indicated by its distinct signaling compared to mature IGF-I signaling, and this concept is discussed in the next section.

### IGF-IR signaling in PCa: Evidence for a novel Ec peptide signaling

According to the IGF signaling models, effective binding of the IGF-I ligand to IGF-IR leads to the activation of signaling pathways that contribute up to 50% of cell growth and proliferation (Baserga et al., 2003). Moreover, ligand activation of the IGF-IR results in a variety of biological effects, including mitogenesis, cell survival, and transformation (Baserga, 1999; Yu and Rohan, 2000; Krueckl et al., 2004; Papageorgiou et al., 2007, 2008). Different domains in the IGF-IR are required for specific functions such as mitogenesis, cell differentiation, transformation, and survival. Thus, TK domain is necessary and sufficient to promote mitogenesis, with lysine 1003 being required for any function of IGF-IR; tyrosine Y950 domain and C-terminus of the receptor are necessary for cell differentiation, while all receptor domains are required for anchorage-independence and transformation. For cell survival, the tyrosine kinase domain, Y950 and a third domain, which resides in a serine quartet at 1280–1283 and binds 14.3.3, are involved (Baserga, 2000).

**Figure 3:**
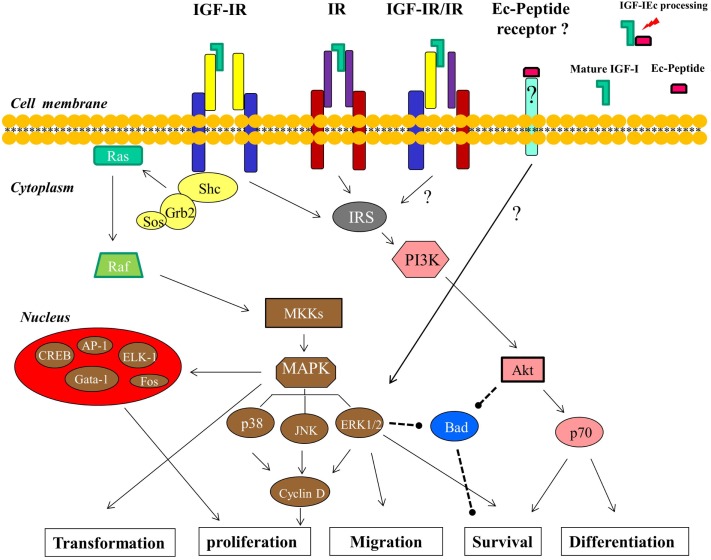
**Signaling pathways and the cellular processes downstream of IGF-I are shown (described in detail in the text)**. The IGF-IR- and IR-independent signaling of the Ec peptide, mediated by a putative Ec peptide receptor, which in its turn can activate ERK1/2, is postulated. The arrows indicate an activating effect and the dashed lines represent an inhibitory effect.

More specifically, phosphorylated IGF-IR activates signaling adaptor proteins including IRS-1, IRS-2, and Src homology/collagen (Shc) (Figure 3); the recruitment of IRS-1 is primarily required for mitogenic signaling, and IRS-2 plays a key role in cellular motility responses (Byron et al., 2006; Ozkan, 2011). Particularly for cell survival, three different pathways, originating from different domains of the IGF-IR, are used. The tyrosine kinase domain acts through the IRS-1/PI3K/Akt/p70 pathway, the second domain (preponderantly Y950) activates Shc and leads to the activation of MAPK pathway, while the third domain activates Raf (Figure 3). The operation of any two of these three pathways is sufficient to protect cells from apoptosis (Peruzzi et al., 1999; Baserga, 2000). However, the primary cell survival pathway activated by IGF-I is the PI3K/Akt signaling pathway (Papageorgiou et al., 2008; Ozkan, 2011). Phosphorylation of PI3K activates the Akt pathway and inhibition of PI3K signaling would prevent the completion of the cell cycle, leading potentially to cell apoptosis or differentiation. However, there is evidence indicated that PI3K inhibition can be overcome by Akt-independent mechanism(s) of protection from apoptosis in PCa cells (Carson et al., 1999). Akt is a kinase activating molecule that causes the induction of anti-apoptotic proteins (Meinbach and Lokeshwar, 2006) and it blocks apoptosis also by phosphorylating, and thus deactivating, the pro-apoptotic Bad protein, a member of the Bcl-2 family of proteins (Moschos and Mantzoros, 2002; Reynolds and Kyprianou, 2006). Bad protein is also a downstream target of the Ras/MAPK/ERK pathway, which is activated by IGF-I and leads both to cell survival and proliferation (Moschos and Mantzoros, 2002; Tenta et al., 2005a; Balmanno and Cook, 2009) (Figure 3).

The Ras/Raf/MEK/MAPK is the second principal pathway associated with activation of IGF-IR. This downstream cascade includes the activation of the small G protein Ras followed by the activation of protein serine kinase Raf, which in turn activates the MEK/MAPK pathway (Figure 3). The final products of this pathway modulate cell proliferation and differentiation via transduction of mitogenic signals through activation of transcription factors (Davis, 1995; Papatsoris et al., 2007; Ozkan, 2011), such as ELK-1, CREB, Gata-1, Fos (Garcia et al., 2006; Steelman et al., 2011), and AP-1 (Kajanne et al., 2009). At the cellular level, IGF-IR increases DNA synthesis and stimulates, through activation of MAPK pathway, the expression of cyclin D1, which accelerates the progression of the cell cycle from G1 to S phase (Furlanetto et al., 1994; Yu and Rohan, 2000; Papatsoris et al., 2007) (Figure 3).

More specifically, MAPKs are a family of serine/threonine protein kinases and are activated in a variety of transformed cells, while the MAPK pathway also mediates, if not totally, cell transformation induced by Ras, Raf, and other oncoproteins (Bodart, 2010). MAP kinases are key mediators of eukaryotic transcriptional responses to extracellular signals and control gene expression via the phosphorylation and regulation of co-regulatory proteins and transcription factors (Papatsoris and Papavassiliou, 2001; Papatsoris et al., 2007; Whitmarsh, 2007; Kaminska et al., 2009; Kim and Choi, 2010). Specifically, one of the pathways that leads from growth factor receptor tyrosine kinases to MAP kinases involves Shc, Grb2, Sos, Ras, Raf, and MKKs; Shc binds to phosphorylated tyrosines on activated IGF-IR tyrosine kinases and the subsequent phosphorylation of Shc generates a binding site for the Grb2, while this binding is thought to generate a Shc/Grb2/Sos complex. Sos is a guanine nucleotide exchange factor that activates Ras which is located at the plasma membrane and in its turn associates with and activates Raf (Crews and Erikson, 1993). The activation of Ras/Raf signals to MKKs (specifically MEK1/2) and these kinases phosphorylate and activate ERKs (ERK1/2), which then can activate by phosphorylation both, other protein kinases and several transcription factors (Figure 3). Ras mediates the activation of various effector pathways that modulate cell proliferation, apoptosis, and other cellular processes (Papatsoris et al., 2007), while further, the ability of the Ras/MAPK pathway to regulate cell proliferation, differentiation, and survival in prostate appears to be dependent upon the amplitude and duration of the MAPK activation (Maroni et al., 2004; Papatsoris et al., 2007). MAP kinase cascades include three major groups of signaling cascades in humans, namely ERK1/2, JNK, and p38 cascade. The ERK cascade is a highly conserved signaling pathway throughout eukaryotic cells, integrating signals that modulate many cellular processes such as cell cycle, proliferation, survival, differentiation, and cell migration (Bodart, 2010). Upon their activation (phosphorylation), ERK1/2 phosphorylate transcription factors present in the cytoplasm or nucleus, thus leading to expression of target genes and resulting in biological responses (Papatsoris and Papavassiliou, 2001; Papatsoris et al., 2007; Kaminska et al., 2009). In this context, the ERK signaling pathway also plays a role in several other steps of tumor development, including the cancer cell migration and tumor invasion, by inducing the expression of matrix metalloproteinases and thereby promoting the degradation of extracellular matrix proteins (Kim and Choi, 2010). The effects of phosphorylated ERK1/2 on PCa cells can be particularly the enhancement of cellular proliferation as well a reduction of apoptosis and, thus, the relative activation of ERK1 and ERK2 could have variable cellular effects in prostate carcinogenesis (Papatsoris et al., 2007). However, growth, survival, or androgen responsiveness of PCa cells are not exclusively mediated by the Ras/MAPK cascade and other molecular mechanisms also converge to this pathway (Papatsoris et al., 2007).

Obviously, the ERK1/2 as well as the Akt signaling pathway, both associated with the ligation of IGF-I to IGF-IR, are involved in PCa development and progression, regulating mainly mitogenic and anti-apoptotic signaling (Papatsoris et al., 2005; Papageorgiou et al., 2007, 2008; Pitulis et al., 2009). Interestingly, however, a distinct signaling of the synthetic human Ec peptide compared to IGF-I ligand (mature peptide) signaling, previously revealed in myoblast-like cells (Philippou et al., 2009) and myocardial-like cells (Stavropoulou et al., 2009), was demonstrated in PC-3 and LNCaP PCa cells (Armakolas et al., 2010). While exogenous administration of the synthetic Ec peptide did activate ERK1/2, it did not activate Akt, suggesting that its mode of action may be different compared with that of mature IGF-I (Armakolas et al., 2010). Moreover, this distinct activation pattern induced by the synthetic Ec peptide was not suppressed after the silencing of either IGF-IR or IR, which are both signaling molecules upstream of ERK1/2 and Akt activation (Kojima et al., 2009). These findings further suggest a bioactivity of human Ec domain in PCa cells which is possibly mediated via an autonomous, IGF-IR- and IR-independent mechanism (Figure 3).

Collectively, it would be of great interest to further confirm the growing body of evidence that the IGF-I mRNA isoforms result in an E domain-specific bioactivity in the pathophysiology of cancer, whether uniquely or in combination with that of the mature IGF-I, and to determine the signaling pathways through which they exert such activity. Given the critical role of IGF-I in cancer, such putative activities of IGF-I Ec peptide could comprise an important potential for illuminating the mechanisms of controlling PCa development and/or progression and for defining candidate targets for therapeutic intervention.

## Concluding Remarks

The important role of the IGF-I bioregulation system in the pathophysiology of PCa is well established, as it regulates various cellular processes such as cell proliferation, differentiation, migration/invasion, and survival. In particular, there is an extensive body of evidence suggesting that the IGFs/IGFBPs/IR system is importantly involved not only in prostate gland growth and development but also in PCa initiation and progression. Due to alternative splicing of the igf-1 gene, different IGF-I mRNA isoforms are produced and a growing interest has arisen with regard to the aspect of Ec products in various pathologies. More specifically, a shift to the up-regulation of the IGF-IEc isoform has been observed, along with other components of the IGF-I system, during the development and progression of PCa, both in *in vivo* and *in vitro* models, implying possible distinct roles of the IGF-I mRNA isoforms in the pathophysiology of the disease. Moreover, a synthetic peptide similar to a C-terminal part of the human Ec domain was shown to possess mitogenic bioactivity in PCa cells and exhibit a distinct signaling pathway as compared to mature IGF-I. It remains a challenge to identify the mechanisms that modulate the IGF-I mRNA isoforms expression and processing during the progression of PCa and to determine whether the PCa cellular responses are regulated both by IGF-I-dependent and Ec peptide-dependent mechanisms.

## Conflict of Interest Statement

The authors declare that the research was conducted in the absence of any commercial or financial relationships that could be construed as a potential conflict of interest.
